# Atualização sobre os tratamentos não cirúrgicos para a dor lombar

**DOI:** 10.1055/s-0045-1810405

**Published:** 2025-11-04

**Authors:** André Wan Wen Tsai, Márcio Fin, Ibrahim Afrânio Willi Liu, Rosana Fontana, Sérgio Mendonça Melo Junior, Jose Eduardo Nogueira Forni

**Affiliations:** 1 Hospital das Clínicas da Faculdade de Medicina da Universidade de São Paulo, São Paulo, SP, Brasil; 2 Clínica Dorto, Sete Lagoas, MG, Brasil; 3 Clínica de Dor, Hospital Madre Teresa, Belo Horizonte, MG, Brasil; 4 Plural Clínica da Dor, Novo Hamburgo, RS, Brasil; 5 Cure Centro Clínico, Morrinhos, GO, Brasil; 6 Faculdade de Medicina de São José do Rio Preto, São José do Rio Preto, SP, Brasil

**Keywords:** analgesia, dor lombar, dor musculoesquelética, tratamento conservador, tratamento multimodal, analgesia, conservative treatment, low back pain, multimodal treatment, musculoskeletal pain

## Abstract

A dor lombar (DL) é uma condição clínica muito prevalente no mundo. Cerca de 90% dos casos são classificados como lombalgias inespecíficas, pois não há alterações anatomopatológicas que justifiquem sua dor. Para uma abordagem terapêutica mais assertiva, é preciso identificar as bandeiras vermelhas ou amarelas, bem como diagnosticar o padrão de dor (nociceptiva, neuropática, nociplástica ou mista) desses pacientes. Nas lombalgias agudas, a evolução natural é favorável na maioria das vezes, e o objetivo do tratamento é evitar a sua cronificação. Nos casos das lombalgias crônicas, visamos a diminuição da dor, e a melhora da funcionalidade e da qualidade de vida. Tanto nos casos agudos quanto crônicos, guiamos nossas condutas pelo princípio da analgesia multimodal, que combina medidas farmacológicas, não farmacológicas e intervencionistas contra a dor. Entre os medicamentos mais utilizados estão analgésicos simples, como o paracetamol, relaxantes musculares, anti-inflamatórios não esteroidais (AINEs) e os analgésicos opioides. Os AINEs e os opioides, quando indicados, devem ser prescritos na menor dose e pelo menor tempo possível. As medicações adjuvantes estão indicadas na presença do componente neuropático. Os fitocanabinoides são possibilidade quando as drogas anteriores falham. Medidas físicas, como calor, laser e terapia de ondas de choque extracorpórea, melhoram a circulação local, produzem relaxamento muscular e tratam o componente miofascial. Intervenções como acupuntura e radiofrequência promovem neuromodulação periférica e/ou central. Alinhar a expectativa do paciente aos resultados dos tratamentos propostos é fundamental; para isso, devemos considerar as medidas educacionais, terapias comportamentais e a reabilitação física.

## Abstract (en)

Low back pain (LBP) is a very prevalent clinical condition worldwide. Approximately 90% of the cases are of nonspecific LBP, due to the lack of anatomopathological changes as potential causes of pain. A more assertive therapeutic approach requires identifying red or yellow flags and diagnosing the pain pattern (nociceptive, neuropathic, nociplastic, or mixed pain). Acute LBP prognosis is favorable in most cases, and the therapeutic objective is to prevent chronicity. For chronic LBP, the goals include pain reduction and improvements in functionality and quality of life. In acute and chronic cases, the principle of multimodal analgesia guides the treatment, which combines pharmacological, non-pharmacological, and interventional methods. The most common medications for LBP are simple analgesics, such as paracetamol, muscle relaxants, nonsteroidal anti-inflammatory drugs (NSAIDs), and opioid analgesics. Opioids and NSAIDs should be prescribed at the lowest dose and for the shortest possible time. Patients with neuropathic components may receive adjuvant drugs. Phytocannabinoids may play a role when the previous pharmacological treatment fails. Physical methods, including heat, laser, and extracorporeal shock wave therapy, improve local circulation, produce muscle relaxation, and treat the myofascial component. Interventions such as acupuncture and radiofrequency promote peripheral and/or central neuromodulation. Aligning the patient's expectations to the outcomes of the proposed treatments is essential; to do so, we must consider the educational measures, behavioral therapies, and physical rehabilitation.

## Introdução

A dor lombar (DL) é uma das causas mais comuns de procura médica no mundo; afeta boa parte da população em idade produtiva, e é a causa mais comum de limitação de atividade física e absenteísmo no trabalho. 1 A prevalência de DL na população americana é de 10 a 30%, sendo que 65 a 80% da população terá lombalgia em algum momento de sua vida. 1 No Brasil, a incidência de DL dentro de 1 ano ultrapassa 50% na população adulta, e a de sua forma crônica varia de 4.2 a 14.7%, dependendo de fatores como nível de escolaridade do indivíduo, índice socioeconômico da região, grau de obesidade e sedentarismo. 2

A etiologia precisa da DL raramente é identificada, uma vez que a maior parte dos quadros são de DL inespecífica, na qual não há presença de alteração anatomopatológica, como compressão radicular ou doença sistêmica. 3 Pacientes com sinais e sintomas de alarme que possam sugerir infecção, malignidade, fraturas, compressão medular e/ou radicular, preenchem critérios para as ditas bandeiras vermelhas da DL, e devem ser abordados de maneira apropriada, com exames laboratoriais e de imagem. 4 Além das bandeiras vermelhas, é importante avaliar os fatores psicológicos e emocionais, como ansiedade, depressão, catastrofismo, cinesiofobia e insatisfações profissionais e pessoais, que são fatores preditivos para a cronificação da DL (bandeiras amarelas). 5

Além disso, é fundamental identificar os diferentes componentes fisiopatológicos que envolvem a dor (nociceptiva, neuropática ou nociplástica), que frequentemente se apresentam como quadro misto nas lombalgias. 6 7

De uma forma geral, a maioria dos casos de DL pode ser manejado com medidas simples e sem a realização de exames complementares. Os pacientes devem ser estimulados a retomar suas atividades assim que a dor permitir. A abordagem terapêutica segue o princípio da analgesia multimodal, e podemos dividi-lo em tratamento farmacológico, não farmacológico (meios físicos, educação e reabilitação) e intervencionistas. 8

### Tratamento Farmacológico

As diretrizes de tratamento farmacológico da lombalgia vêm de estudos 9 com muitas limitações, como ensaios de curto prazo e aqueles que envolvem populações heterogêneas. Os medicamentos orais mais comumente prescritos para a lombalgia incluem paracetamol e anti-inflamatórios não esteroides (AINEs), relaxantes musculares, antidepressivos, anticonvulsivantes e opioides. 9 Esses medicamentos têm eficácia muito similar na redução da dor, e estão associados a diferentes efeitos colaterais. Independentemente da classe, as evidências 9 apontam para uma boa eficácia da analgesia no curto prazo e com boa segurança; no entanto, sua eficácia no longo prazo não está clara.

A indicação medicamentosa varia com relação aos quadros agudos (< 6 semanas de duração), subagudos (6–12 semanas de duração) e crônicos (> 12 semanas de duração). Faz-se uso dos AINES em todas as fases, e ao mesmo tempo observamos a diminuição do uso de opioides e aumento do uso de antidepressivos e antiepilépticos com a evolução da cronificação 10 ( Tabela 1 ).

**Tabela 1:** Prevalência de indicação das classes farmacológicas com base no tempo de evolução da lombalgia 10

N: 22	AINEs	Relaxantes musculares	Opioides	Paracetamol	Antidepressivos	Anticonvulsivantes
Aguda	54,5%	27,2%	36,3%	22,7%	9,1%	4,5%
Subaguda	50%	22,7%	18,2%	18,2%	9,1%	4,5%
Crônica	59,1%	22,7%	27,2%	18,2%	27,2%	13,6%
Não classificada	18,2%	NAV	13,6%	9,1%	4,5%	NAV

**Abreviaturas:** AINEs, anti-inflamatórios não esteroidais; N, número de diretrizes; NAV, não avaliados.

### AINES e Analgésicos Simples

As evidências sugerem um curso curto de paracetamol ou AINEs para o tratamento da DL, e sua utilização prolongada deve ser evitada. 9 Uma revisão sistemática 9 não identificou diferenças significativas entre o paracetamol e os AINEs no alívio da DL. Porém, diferentemente dos AINEs, o paracetamol não está associado ao aumento do risco de infarto do miocárdio ou sangramento gastrointestinal, e é uma opção mais segura para pacientes com predisposição a essas condições. 9

A dipirona, ou metamizol, é amplamente utilizada no Brasil, apesar de haver pouca evidência na literatura mundial que sustente o seu uso na DL. No entanto, a Sociedade Brasileira de Reumatologia 11 a recomenda para o tratamento da lombalgia.

Em uma revisão sistemática com metanálise, Wewege et al. 12 relataram que a confiança nas evidências sobre a redução da intensidade da dor com vários AINEs e analgésicos era baixa ou muito baixa. Medicamentos como tolperisona, aceclofenaco com tizanidina e pregabalina mostraram alguma eficácia, mas com incertezas significativas. Além disso, a análise 12 indicou que alguns medicamentos, como tramadol e combinações de paracetamol com tramadol, estavam associados a um aumento moderado do risco de eventos adversos.

### Relaxantes Musculares

Os relaxantes musculares são classificados em dois grupos: 9

- Antiespásticos: baclofeno, tizanidina, dantrolene e diazepam. Não são recomendados para DL não específica, e são indicados principalmente para o tratamento da espasticidade associada a doenças do sistema nervoso central, como esclerose múltipla. Devido ao seu potencial de dependência, os benzodiazepínicos devem ser evitados. 8 9
- Antiespasmódicos: ciclobenzaprina e carisoprodol. Podem ser usados em curto prazo (2 semanas) em quadros agudos, e seu uso prolongado não é recomendado. 8 9

### Antidepressivos

Os antidepressivos tricíclicos (amitriptilina e nortriptilina) demonstraram uma pequena redução da dor em pacientes com lombalgia crônica. Já os inibidores seletivos de recaptação de serotonina não demonstraram eficácia superior à do placebo na fase crônica da dor. Por outro lado, os inibidores de recaptação da serotonina-norepinefrina (venlafaxina e duloxetina), demonstraram potencial analgésico para determinadas condições. 9 Os antidepressivos tricíclicos, a duloxetina e a venlafaxina são considerados medicamentos de primeira linha de tratamento da dor neuropática. 13

### Anticonvulsivantes

A gabapentina pode ser indicada para lombalgia crônica em determinados grupos de pacientes, como aqueles com estenose espinhal, claudicação neurogênica e dor radicular, especialmente quando não há resposta satisfatória aos tratamentos iniciais. Outros anticonvulsivantes, como carbamazepina, pregabalina e lamotrigina, não são recomendados formalmente devido à evidência limitada. No entanto, em pacientes com dor neuropática, a gabapentina é considerada tratamento de primeira linha, ao passo que a pregabalina é classificada como de segunda linha. 13

**Fig. 1:**
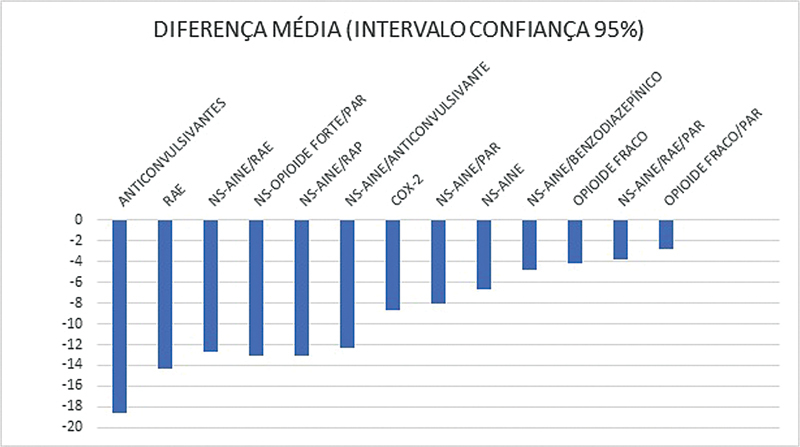
Medida do efeito analgésico das classes medicamentosas em comparação ao placebo. Quanto mais à direita, mais favorável ao placebo. **Abreviaturas:** RAE, relaxante muscular antiespasmódico; NS, não seletivo; PAR, paracetamol; COX-2, inibidores de ciclo-oxigenase 2; RAP, relaxante muscular antiespástico. 12

Uma revisão sistemática 12 indicou que os anticonvulsivantes podem estar associados a reduções moderadas na intensidade da dor em comparação com o placebo, ainda que a confiança na evidência seja considerada muito baixa. Mesmo assim, demonstraram superioridade em relações a outras classes terapêuticas 12 ( Fig. 1 ).

### Opioides

A terapia de curto prazo com opioides pode ser indicada para o manejo da lombalgia aguda grave, com respaldo na literatura. No entanto, o uso prolongado deve ser realizado com cautela e supervisão rigorosa, visando à reabilitação do paciente e ao cumprimento de objetivos terapêuticos bem definidos. A descontinuação do tratamento deve ser considerada em casos de falha terapêutica ou comportamento de risco repetido, como transtorno de uso de substâncias. 9 Observa-se uma tendência à redução da prescrição de opioides conforme a lombalgia se torna crônica. 10

### Cannabis

Os fitocanabinoides modulam as vias de dor por meio da ativação dos receptores canabinoides. Embora os mecanismos farmacológicos responsáveis pelos efeitos analgésicos ainda não estejam totalmente esclarecidos, pesquisas recentes têm destacado as propriedades anti-inflamatórias dos fitocanabinoides e seu impacto no alívio da dor. A literatura sobre a eficácia da cannabis como analgésico é vasta. 14 Uma revisão controlada por placebo 15 concluiu que há evidência de qualidade moderada que apoia o uso de canabinoides para o tratamento da dor crônica.

Os receptores canabinoides estão amplamente distribuídos nas vias de modulação da dor, incluindo neurônios sensitivos periféricos e centrais, e também influenciam a regulação das respostas emocionais aos estímulos nocivos e de dor. No nível supraespinhal, esses receptores modulam os componentes cognitivo e emocional da percepção da dor no córtex sensitivo-motor e na amígdala, o que reduz as sensações desagradáveis associadas à dor. Na periferia, a ativação dos receptores canabinoides do tipo 1 (CB1) inibe a transmissão dos estímulos nociceptivos, ao passo que a ativação dos receptores canabinoides do tipo 2 (CB2), localizados nas células imunes da pele, inibe a liberação de citocinas inflamatórias. 16

### Biológicos

O envolvimento do fator de necrose tumoral alpha (FNT-α) na herniação do disco, na sensibilização nervosa e no crescimento interno é respaldado por estudos em modelos animais, que demonstram efeitos preventivos do bloqueio desse fator inflamatório. A produção local do FNT-α encontra-se aumentada em discos herniados e degenerados, o que motivou estudos clínicos 17 para avaliar a eficácia da inibição no tratamento da dor discogênica em humanos. Os agentes biológicos, ao bloquearem ou inibirem etapas cruciais na geração e na propagação da inflamação, podem desempenhar um papel complementar no manejo da DL baixa e da ciática, especialmente quando as intervenções tradicionais fracassam. No entanto, apesar de resultados promissores em estudos pré-clínicos, as evidências clínicas ainda são inconsistentes. Duas classes de biológicos foram testadas: 17 anti-FNTs em pacientes com DL baixa crônica com radiculopatia e anti-fator de crescimento nervoso (anti-FCN) para DL crônica não radicular. A qualidade das revisões sistemáticas é baixa devido à heterogeneidade dos estudos. Além disso, poucos trabalhos testam essas terapias diretamente com outras opções farmacológicas, o que torna necessária a realização de mais estudos. Na DL crônica inespecífica, o tanezumabe pode ser mais eficaz no controle da dor e na melhora funcional. No entanto, ao considerar os anti-FCNs como uma classe de fármacos, os resultados não são tão consistentes, e não sustentam uma recomendação para uso na DL sem radiculopatia, dado que as evidências ainda são frágeis. 17

### Medidas Físicas

O calor e a crioterapia superficial promovem um aumento inicial da circulação sanguínea, seguido de um resfriamento local, o que pode aliviar o tensionamento muscular e melhorar a restrição do movimento articular. Essas técnicas demonstram eficácia moderada na redução da dor e da incapacidade em quadros de DL aguda e subaguda no curto prazo, especialmente quando associados a exercícios físicos. No entanto, a maioria dos estudos são de evidência limitada. Para a DL crônica, as evidências sobre a eficácia da termoterapia de adição (calor) e da crioterapia são insuficientes, e conflitantes quanto à avaliação da diferença entre ambas. 6 18

O ultrassom terapêutico (UST) não apresentou eficácia no tratamento da DL. Evidências de baixa qualidade 19 não conseguiram demonstrar as diferenças entre o ultrassom real e o simulado para a redução da dor após o tratamento no seguimento de quatro semanas, assim como em comparação ao grupo controle. Além disso, não foram observados efeitos significativos na melhora da função. No médio e longo prazo, as evidências permanecem insuficientes. 19

Na estimulação elétrica nervosa transcutânea (EENT), as evidências também são insuficientes para comprovar eficácia no tratamento da DL crônica. Estudos de baixo nível de evidência 20 21 não identificaram diferenças entre EENT real e simulada na redução da intensidade da dor ou no ganho de função no curto prazo.

Já a fotobiomodulação com laser de baixa intensidade (FLBI) consiste na aplicação de luz em diferentes comprimentos de onda sobre a pele, o que promove reações bioquímicas que buscam reduzir a inflamação local e aliviar a dor. Para a DL, evidências de baixa qualidade 22 23 sugerem um pequeno a moderado efeito de melhora no curto prazo na dor e um benefício discreto na função.

Já a fotobiomodulação com laser de alta intensidade (FLAI) demonstrou, em uma meta-análise, 22 uma redução significativamente maior da dor em comparação ao grupo controle, além das melhorias nos índices de incapacidade de Oswestry e de Roland-Morris. Em um ensaio clínico prospectivo 23 que comparou FLAI com UST, o grupo tratado com FLAI apresentou uma redução estatisticamente significativa da dor e da incapacidade após o tratamento e no seguimento de três meses.

O tratamento por ondas de choque extracorpóreas (TOCE) utiliza ondas mecânicas de alta pressão e curta duração, seguidas de uma fase com pressão negativa. Esse processo desencadeia um fenômeno de mecanotransdução, que resulta em efeitos biológicos sobre o tecido afetado, com o objetivo de analgesia, aumento da vascularização local, regeneração tecidual e osteogênese. Estudos indicam que o TOCE apresenta efeitos positivos sobre a síndrome miofascial, pois contribui para a regularização da matriz extracelular. Em um estudo prospectivo randomizado, 24 o TOCE associado a um programa de exercícios demonstrou redução da DL crônica em comparação ao placebo (apenas exercícios) dentro de 3 meses. No entanto, não houve impacto significativo na função.

As evidências disponíveis 19 não foram suficientes para comprovar a eficácia de outros métodos físicos, como estimulação elétrica muscular, diatermia de ondas curtas, tanto no controle da DL aguda e subaguda, como crônica, com ou sem radiculopatia.

### Métodos Intervencionistas

Os métodos intervencionistas são procedimentos médicos utilizados para alívio da dor e/ou auxílio diagnóstico (bloqueio teste), geralmente realizados sob orientação por imagem. 25

Além dos tratamentos clínicos mencionados neste artigo, intervenções específicas podem ser consideradas com base na estrutura envolvida na lombalgia inespecífica: 25

a) Componente discogênico – mais comum em pacientes mais jovens, geralmente com idades abaixo de 40 anos, caracteriza-se por dor exacerbada na flexão do tronco;
b) Componente facetário – mais frequente em pacientes idosos, com idades acima de 60 anos, que apresentam dor na hiperextensão da coluna lombar;
c) Componente nas articulações sacrilíacas – mais prevalente em pacientes submetidos a artrodese da coluna lombar com piora da dor ao permanecer sentado;
d) Componente miofascial – altamente prevalente em dores musculoesqueléticas, responde bem a tratamentos como acupuntura.

### Acupuntura

A acupuntura é um tratamento médico amplamente difundido no mundo devido à sua eficácia clínica comprovada para diversas condições, especialmente dores aguda e crônica, incluindo a DL. 19 26 27 Alguns mecanismos envolvidos na analgesia da acupuntura já estão bem estabelecidos, como a redução de citocinas pró-inflamatórias no ponto agulhado, a modulação no nível do corno posterior da medula espinal e a ativação do sistema inibitório descendente tanto à via serotoninérgica quanto à via noradrenérgica. 27 Além disso, o acionamento do eixo hipotálamo-hipófise-adrenal e o envolvimento do sistema opioidérgico endógeno também estão bem estabelecidos. Outros mecanismos ainda estão em estudo, 27 incluindo a inativação de pontos gatilhos, o sistema endocanabinoide, a degranulação de mastócitos e a transdução por meio de receptores de adenosina A1, canal catiônico de potencial transitório do receptor, subfamília V, membro 1 ( *transient receptor potential cation channel subfamily V member 1* , TRPV1, em inglês) e canal catiônico de potencial transitório do receptor, subfamília V, membro 2 ( *transient receptor potential cation channel subfamily V member 2* , TRPV2, em inglês). Diversos estudos 28 29 30 demostram melhora da dor e função na DL em comparação ao grupo placebo. O alívio da dor e a melhora da função são observados em média após 5 sessões, sendo que os melhores resultados ocorrem quando a acupuntura é associada ao tratamento convencional, seja ele medicamentoso ou não. 31

### Radiofrequência e Outros Bloqueios

A denervação por radiofrequência é um método invasivo utilizado no tratamento de lombalgia crônica inespecífica, e pode ser indicado quando a origem da dor é um disco, uma faceta ou a articulação sacrilíaca. Embora haja controvérsia em relação aos resultados, é mais um instrumento no controle da dor. Apesar da ausência de evidências científicas robustas sobre a duração do alívio da dor, os principais especialistas 32 a recomendam como uma alternativa adicional no manejo da DL crônica.

As principais intervenções utilizadas no tratamento das lombalgias incluem: bloqueios facetários, bloqueio discogênico, bloqueio da articulação sacrilíaca, radiofrequência pulsada e ablativa de facetas e bloqueio do ramo médio.

Dor lombar de origem discogênica: após a confirmação do diagnóstico por exame físico, exames de imagem e comprovação por discografia provocativa, a DL de origem discogênica pode ser tratada com radiofrequência pulsada intradiscal, e bons resultados foram descritos na literatura internacional. 33

Dor lombar de origem facetária: após o diagnóstico clínico, exames de imagem e confirmação por bloqueio teste injetando 0,5 mL de xilocaína a 2% na faceta com taxa de melhora da dor superior a 70%, o tratamento pode ser realizado com radiofrequência ablativa da articulação e radiofrequência pulsada do ramo medial do nervo espinhal. 34 35 36 As radiofrequências ablativas apresentam melhores resultados no longo prazo no controle da dor. 34 35 36

Dor cuja origem é a articulação sacrilíaca: o diagnóstico deve ser confirmado com exame clínico, exames de imagem e bloqueio teste injetando-se 0,5 mL de xilocaína a 2%. Se houver melhora acentuada da dor, o tratamento pode ser realizado com a injeção de dexametasona associada a ropivacaina. 36 Este procedimento tem duração curta no controle da dor. Alternativamente, o bloqueio da sacrilíaca por radiofrequência ablativa ou por crioterapia pode promover uma melhora na dor por um período mais prolongado. 37

### Educação e Reabilitação Física e Mental

A educação sobre dor musculoesquelética tem como objetivo fornecer informações relevantes sobre a dor e a condição clínica, especialmente para aqueles que apresentam maior grau de ansiedade, preocupação, comportamento passivo ou medo. 38 O esclarecimento adequado permite ao paciente uma melhor compreensão da sua dor, bem como das diversas modalidades disponíveis, que podem ajudar a minimizar quadros de cinesiofobia e a catastrofização. Orientar a mudança de estilo de vida visando a perda de peso e a cessação do tabagismo e conscientizar o paciente quanto ao seu protagonismo no processo de tratamento aumentam a taxa de bons resultados. 38

A terapia cognitivo-comportamental (TCC) é a abordagem de dores musculoesqueléticas crônicas, com efeito pequeno a moderado de melhora da dor na DL crônica. O objetivo da TCC é modificar um dos três sistemas de resposta (comportamental, cognitivo e reatividade fisiológica). As intervenções mais promissoras para um tratamento físico e de reabilitação em pacientes com lombalgia crônica são um tratamento multidisciplinar ou tratamento comportamental. Todos os tipos de terapia comportamental foram mais eficazes na redução da intensidade da dor quando comparados ao grupo controle. Além disso, há algumas indicações de que o acréscimo de componentes comportamentais pode reduzir o afastamento do trabalho e os custos devidos à licença médica. 20 39

Uma outra abordagem, a técnica de atenção plena ( *mindfulness* ), que busca diminuir o estresse ao estimular que a mente se concentre no momento presente, pode ter um efeito pequeno a moderado no curto prazo, mas menor no médio prazo, na DL. Um estudo demonstrou que 60% dos pacientes submetidos a essa técnica apresentaram uma melhora de 30% em relação aos valores basais com significância clínica depois de 6 meses. Quando comparado com o grupo da TCC, 58% dos pacientes deste grupo obtiveram melhora, assim como 44% do grupo de cuidados usuais. 19 39 Tanto a TCC quanto a técnica de atenção plena visam ajustar pensamentos e comportamentos que perpetuam a dor. Ambas contribuíram de forma moderada para o ganho funcional, resultado que persistiu por até 2 anos. 19 39

A terapia por exercícios também reduziu significativamente a intensidade da dor e a incapacidade em comparação aos cuidados habituais; há fortes evidências na literatura 20 40 de que exercícios voltados para o fortalecimento da musculatura são responsáveis pela estabilização da coluna e da pelve (os músculos do *core* ).

### Considerações Finais

A DL é condição clínica altamente prevalente no Brasil e no mundo, sendo que causas específicas, como fraturas, hérnias discais, estenose de canal e doenças sistêmicas correspondem a menos de 10% de todos os casos. 10 19 O tratamento farmacológico faz parte do manejo multimodal da lombalgia independentemente da fase clínica, com preferência pelo uso por curto período de tempo, especialmente dos AINES e dos opioides. Algumas drogas, como os gabapentinoides, antidepressivos tricíclicos e duais são a primeira opção em casos de dor neuropática associada a lombalgia. 13

A cannabis surge como uma possibilidade de tratamento quando as medicações convencionais não produzem resultados satisfatórios. Além disso, os métodos intervencionistas no tratamento da lombalgia inespecífica são um recurso adicional que poder ser utilizado pelo médico, como adjuvantes aos outros tratamentos farmacológicos, com o objetivo de permitir uma reabilitação mais rápida e eficaz.

Por fim, não podemos esquecer a importância dos meios físicos, da educação e da reabilitação física e mental tanto para pacientes com lombalgia aguda quanto crônica.
